# Structural and magnetic study of undoped and cobalt doped TiO_2_ nanoparticles

**DOI:** 10.1039/c8ra00626a

**Published:** 2018-03-19

**Authors:** Anupama Chanda, Kumarmani Rout, M. Vasundhara, Shalik Ram Joshi, Jai Singh

**Affiliations:** Department of Physics, Dr Hari Singh Gour Central University Sagar M.P India jai.bhu@gmail.com anupamamatsc@gmail.com; CSIR-National Institute for Interdisciplinary Science and Technology (NIIST) Trivandrum – 695 019 Kerala India mvas@niist.res.in; School of Mechanical, Aerospace and Nuclear Engineering, Ulsan National Institute of Science and Technology(UNIST) UNIST-gil 50 Ulsan 689-798 Republic of Korea

## Abstract

The present study investigates the influence of cobalt doping on the structural and magnetic properties of TiO_2_ nanoparticles prepared by a simple wet chemical method. The single phase anatase structure of Co-doped TiO_2_ nanoparticles was confirmed by X-ray powder diffraction. A morphological study using scanning electron microscopy and transmission electron microscopy indicates the formation of TiO_2_ nanoparticles of sizes 6–10 nm. The high resolution TEM image shows clear lattice fringes indicating the highly crystalline nature of the nanoparticles which was further analysed by selected area electron diffraction pattern which indicates a polycrystalline nature of anatase TiO_2_. The shifting and broadening of the most intense E_g_ (1) mode in micro-Raman study of Co-doped TiO_2_ nanoparticles and XPS spectra indicate the incorporation of Co in TiO_2_. Magnetic measurement shows ferromagnetic behavior at room temperature in undoped TiO_2_ which has originated due to the presence of oxygen vacancies which are intrinsic in nature. But the *M*–*H* curve of Co-doped TiO_2_ shows the coexistence of ferromagnetic and paramagnetic phases with enhanced magnetization. The enhancement in magnetization has arisen due to Co doping and the paramagnetism may be due to the presence of some undetected clusters of oxides of cobalt.

## Abstract 2

The present study investigates the influence of cobalt doping on the structural and magnetic properties of TiO_2_ nanoparticles prepared by a simple wet chemical method.

## Graphical abstract

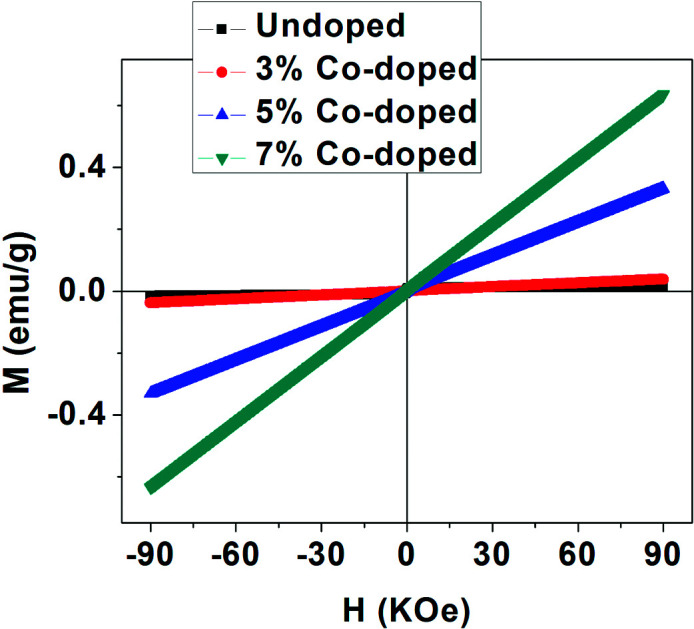

## 1. Introduction

Dilute magnetic semiconductors (DMS)^1^ formed by doping a sizable amount of transition metal into a semiconductor have been of great interest to researchers due to their possible applications in spintronics^2^ devices like spin light-emitting diodes (spin-LEDs), spin field effect transistors (spin-FETs) and spin qubits for quantum computers and spin-based memory devices like MRAM^3–6^ in which both the spin and the charge of the electrons can be used. One of the major challenges for semiconductor spintronics devices is the injection and transport of spin polarized charge carriers. The realization of such DMS is still a widely discussed issue due to the questionable magnetic behavior of the material. For data storage and other device applications ferromagnetism (FM) at room temperature (RT) is one of essential requirements. Although successful doping could be achieved in Mn based III-V semiconductors, but their curie temperature was found unsuitable for practical device applications.^7,8^ Since then a large number of efforts has been carried out to find the possibility of FM at RT in III-V based DMS such as GaN, GaSb, InAs^9–12^ and oxide-based DMS such as ZnO, TiO_2_, SnO_2_, In_2_O_3_*etc*^13–24^.

Nanosized titanium dioxide (TiO_2_) materials have attracted considerable attention due to its modified electronic and optical properties which provide extensive applications in photo-catalysis, sensors, solar cells, spintronics, energy storage, waste water management, as well as for self-cleaning surfaces.^25–30^ It can also be used as an antibacterial agent because of strong oxidation activity and superhydrophilicity.^31^ However due to its wide band gap (3.0–3.2 eV) its activity is limited to near-ultraviolet region. Doping TiO_2_ with transition metals tunes the electronic structure and shifts the light absorption region from UV to visible light as well as it gives FM at RT which can be of potential use in spintronics devices. After the experimental report by Matsumoto^30^ about FM at RT in Co-doped TiO_2_ thin films, much effort has been focussed on TiO_2_ as a host material for magnetic ion doping. Subsequently, extensive research has been carried out on Co-doped TiO_2_ thin films which were grown by different growth techniques like pulsed laser deposition, laser molecular beam epitaxy (LMBE), combinatorial LMBE, sputtering, metal organic chemical vapour deposition as well as sol–gel technique.^32–39^ FM at RT has been observed by many groups in Co-doped TiO_2_ anatase and rutile phases.^40–42^ However the origin of FM in these materials has been of controversial nature like whether the FM arises due to substitution of magnetic ions on the host lattice sites or due to formation of secondary phases of dopant ions. Recently FM in oxide based DMS has been observed as an intrinsic property induced by defects such as oxygen vacancy and titanium vacancy.^43–45^ Karthik *et al.*^46^ reported FM at RT in Co-doped TiO_2_ and observed the dependence of magnetic behaviour on dopant concentration while Choudhury *et al.*^28^ reported ferromagnetic coupling between two dopant ions arising due to oxygen vacancy. Santara *et al.*^42^ observed FM at RT in doped system which arises from intrinsic exchange interaction of magnetic moments mediated by defects in doped nanoparticles. Hong *et al.*^47^ and Yoon *et al.*^48^ have claimed that FM in TiO_2_ thin films has been caused by oxygen vacancies while Kim *et al.*^49^ reported oxygen vacancy induced lattice distortion created FM. The spintronics application requires that FM should be intrinsic and it should not arise due to magnetic clusters formed due to transition metal doping. Few studies have been done on Co-doped TiO_2_ nanoparticles (NPs) in contrast to thin films. In this study, undoped and Co-doped anatase TiO_2_ NPs have been prepared by a simple cost effective chemical route whose structural, morphological and magnetic properties have been studied.

## 2. Experimental details

Synthesis of un-doped and Co-doped (3 at%, 5 at%, 7 at%) TiO_2_ NPs were carried out using titanium diisopropoxide bis-(acetylacetonate) (C_16_H_28_O_6_Ti) as the starting materials. For the synthesis of un-doped TiO_2_ NPs, precursor and distilled water were taken to prepare a 1 molar solution to which drop of conc. HNO_3_ was added to maintain the pH of the solution. Then the solution was kept on a magnetic stirrer for duration of 40 minutes. After 40 minutes the solution was heated at a temperature of 50 °C to evaporate the water present in the sample. Once it is completely dried it was crushed into uniform powders using a mortar and pestle. At last the powder was kept inside the heated oven for calcinations for 2 h at 400 °C. For preparation of Co-doped TiO_2_ nanoparticles the same procedure was followed as in the case of pure TiO_2_ along with the addition of requisite amount of cobalt chloride in different concentrations (3%, 5% and 7%) which was added in the precursor in the first step of the procedure described above for the preparation of un-doped TiO_2_. Thereafter the same procedure was followed for the whole preparation process. By adding different concentrations of cobalt chloride, differently doped TiO_2_ NPs were obtained.

The crystal structure and phase purity of the powdered samples were analyzed by X-ray powder diffraction (XRD) technique (PANalytical-Empyrean Diffractometer) using Cu K_α_ source of wavelength 1.5404 Å and scan size of 0.01° from 20° ≤ *θ* ≤ 80°. Rietveld refinement of the diffraction patterns was carried out using the Fullprof software. The morphologies of the NPs were investigated by scanning electron microscope (SEM, Jeol), and transmission electron microscope (TEM, (FEI Tecnai F20, operated at 300 kV)). The crystal planes were found out from selected area electron diffraction (SAED) pattern. Micro-Raman scattering study was carried out with 633 nm line of a He–Ne ion laser at room temperature with a Renishaw Invia Raman microscope. X-ray photoelectron spectroscopy (XPS) spectra was acquired using a PHY 5000 Versa Probe II, ULVAC-PHI, Inc instrument and a Al Kα1 X-ray source at room temperature. Pressure in the XPS chamber during the measurements was 5 × 10^−10^ mbar. The binding energies were corrected by taking C 1s as reference energy (C 1s = 284.8 eV). A wide scan was collected to ensure that no foreign materials were present on the sample surface. The high-resolution scans of Ti 2p and Co 2p regions were collected. Curve fitting to the XPS spectrum was done using MultiPak Spectrum:ESCA. Background subtraction was done using the Shirley method. Magnetic measurements of the samples were made as a function of applied field using a vibrating sample magnetometer attached to a physical property measurement system supplied by Quantum Design Inc., USA.

## 3. Results and discussion

**Fig. 1:**
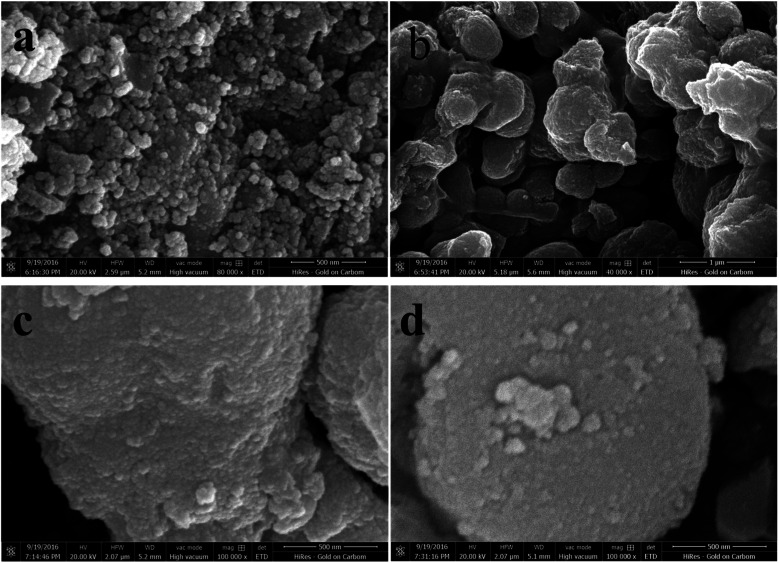
(a) SEM image of TiO_2_, (b) SEM image of 3% Co-TiO_2_, (c) SEM image of 5% Co-TiO_2_, (d) SEM image of 7% Co-TiO_2_.

The morphology of the TiO_2_ powder was studied by SEM and TEM. Fig. 1 shows SEM images (a–d) of un-doped and Co-doped TiO_2_ samples. Spherical shaped nanoparticles can be seen from un-doped sample while agglomeration of particles making big clusters can be seen in Co-doped samples. The small particles are agglomerated and bound to the spherical shape due to the doping of Co. To further illucidate the size and structure of these particles TEM was carried out.

**Fig. 2:**
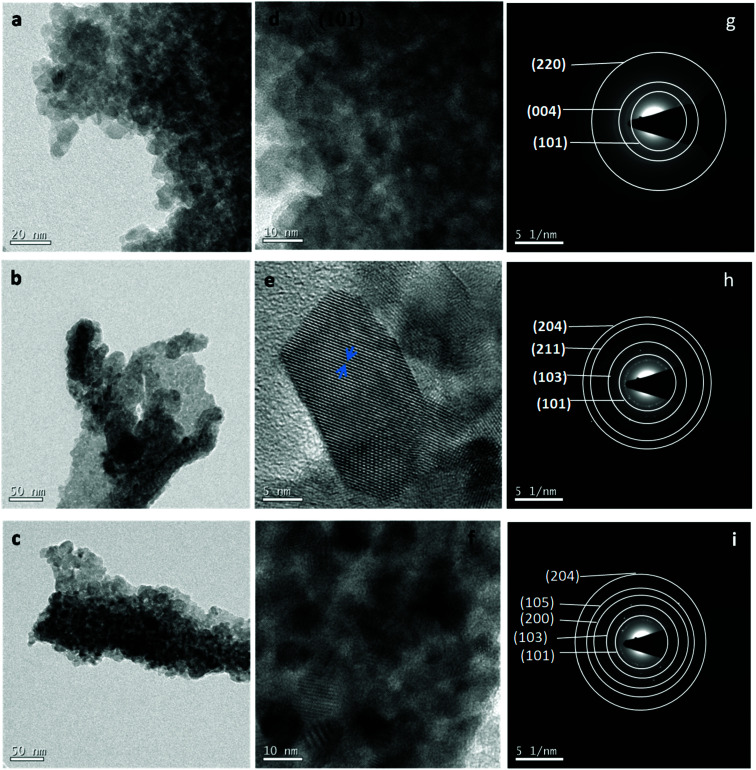
(a–c) TEM image of TiO_2_, 3% Co-TiO_2_ and 5% Co-TiO_2_, (d–f) HRTEM image of TiO_2_, 3% Co-TiO_2_ and 5% Co-TiO_2_, (g–i) SAED pattern of TiO_2_, 3% Co-TiO_2_, 5% Co-TiO_2_.

Fig. 2(a–c) show the TEM images taken on un-doped and Co-doped (3% and 5%) TiO_2_ powder while Fig. 2(d–f) indicate the HRTEM images and 2(g–i) show the SAED pattern taken on un-doped, 3% and 5% Co-doped TiO_2_ NPs. TEM images show almost spherical shaped particles with uniform size distribution. Particle size found out from TEM image is in the range 6–15 nm which is in good agreement with the crystallite size obtained from XRD (discussed later). HRTEM show clear lattice fringes with *d*-spacing of 0.37 nm in undoped, 0.365 nm in 3% and 0.36 nm in 5% doped samples which corresponds (101) plane of tetragonal anatase phase of TiO_2_ indicating the preferable crystal growth plane is (101) which is also the highest intense peak in XRD. Image J software was used to find out the *d*-spacing from HRTEM images. The SAED patterns taken on un-doped and Co-doped samples show clear distinct rings corresponding to different planes of tetragonal anatase TiO_2_ structures. The rings obtained in the SAED pattern indicate the formation of polycrystalline anatase TiO_2_ NPs. The planes corresponding to different rings have been found out by calculating the *d*-spacing using Image-J software which is in agreement with the planes obtained in XRD and those planes correspond to tetragonal anatase phase of TiO_2_.

**Fig. 3:**
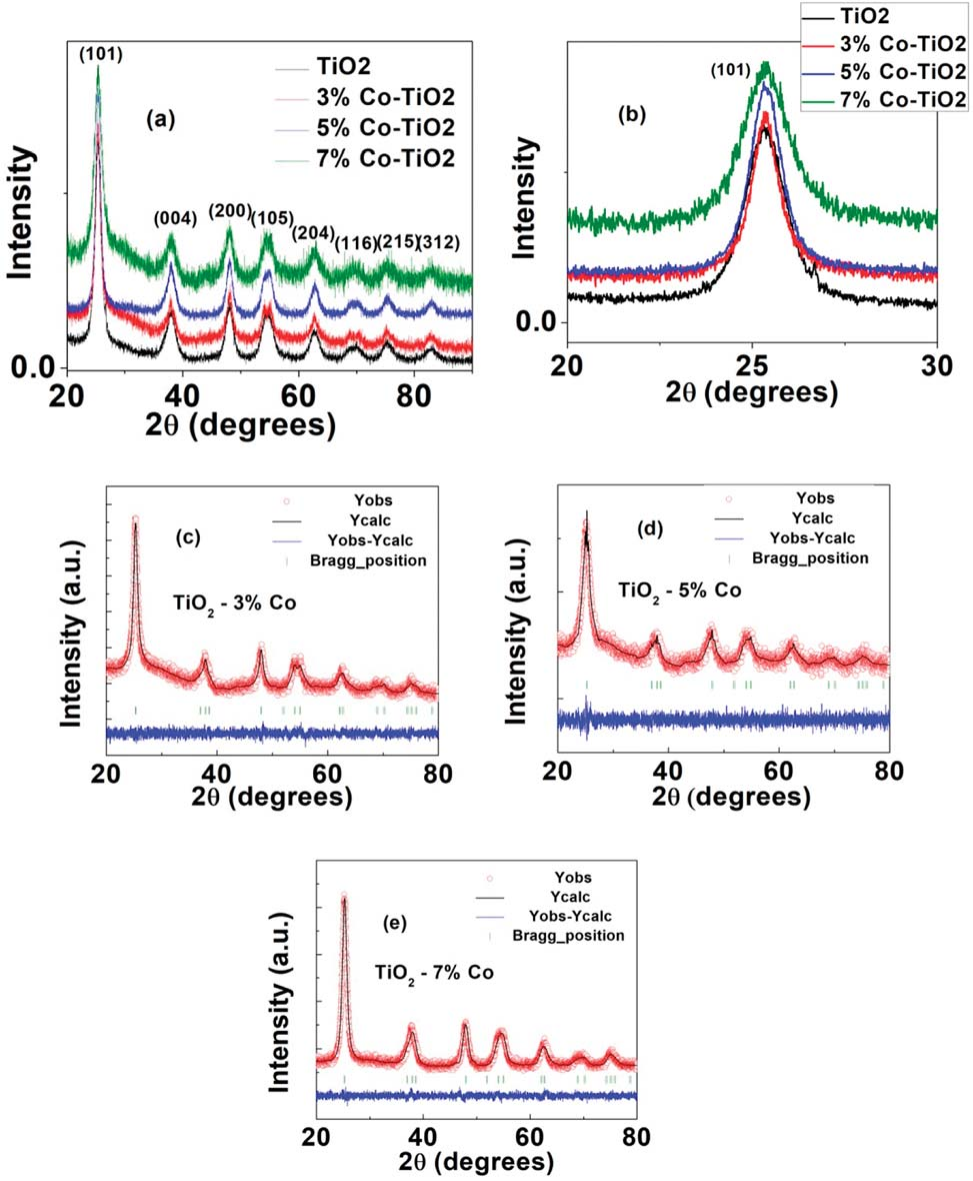
(a) XRD pattern of undoped and Co-doped TiO_2_, (b) expanded region of (101) peak of figure (a) around 20–30 degrees, (c) Rietveld refinement data of 3% Co-TiO_2_, (d) Rietveld refinement data of 5% Co-TiO_2_, (e) Rietveld refinement data of 7% Co-TiO_2_.

The structural parameters and phase purity were studied using powder X-ray diffraction and Full-proof software. The XRD patterns of undoped and Co-doped TiO_2_ powder with varying dopant concentrations from 3% to 7% are shown in Fig. 3a. All the samples are found to crystallize in single anatase phase with a space group *I*4_1_/*amd* (JCPDS 78-2486) without any rutile peak or peaks related to metallic cobalt or cobalt oxide confirming that anatase phase is not disturbed upon Co doping in TiO_2_. The most intense peak (101) (shown in Fig. 3b) shows a slight shifting of the peak position towards higher angle as well as change in FWHM indicating change in local structure around Ti^4+^ after Co doping. This shift in peak position and change in FWHM with cobalt doping (Fig. 3b) indicate the incorporation of Co in TiO_2_.^50^ The particle size of undoped and Co-doped TiO_2_ NPs determined from XRD pattern using Scherer's equation was found out to be 7 nm and 6–11 nm respectively which matches well with the particle size obtained from TEM data. The change in *d*-spacing calculated for (101) peak from undoped to 3%, 5% and 7% doped samples are 0.0019 nm, 0.0036 and 0.0040 nm respectively. As the change in *d*-spacing is not that prominent this means that a very few percentage of Co has taken part in substituting Ti^4+^ and others may have gone to interstitial site or in grain boundary or on the surface. As the ionic charge of Ti (+4) and Co (+2) are different, dopant substitution leads to creation of oxygen vacancy to balance the charge neutrality. This vacancy as well as dopant atoms at the grain boundary disturb the lattice structure and thereby change the crystallinity with doping. For further understanding, Rietveld refinement of the data was done which are shown in Fig. 3(c–e) and the refined parameters are given in Table 1. The Rietveld refinement confirms that TiO_2_ crystallizes in the anatase tetragonal structure and presence of no secondary phase has been detected due to Co-doping. From the refined data, it can be seen that there is slight change (increase) in lattice parameter in cobalt doped samples which is expected as the ionic radius of Co^2+^ (0.65 Å) is larger than that of Ti^4+^ ions (0.61 Å).^51^ The changes in “*a*” and “*c*” lattice parameters are observed due to the incorporation of Co dopant, as shown in Table 1. It can be seen from the table that there is increase in lattice parameter “*a*” with increase in cobalt except in 3% doped sample where lattice parameter is decreased very little (4^th^ decimal point). Similarly the variation in “*c*” axis lattice parameter is in increasing trend upto 5% doped sample and in 7% doped sample it has decreased. But it can be seen from the table that the volume of the unit cell increases with the increase of Co doping level although there is very small variation in a and *c* axis parameter. The difference between ionic radii of the dopant and host ions is expected to cause a small enhancement of the TiO_2_ unit cell. According to Vegard's law, higher doping levels could increase the volume of the unit cell.^52^

**Table 1:** Rietveld refinement of powder XRD data of undoped and Co-doped TiO_2_ (T1: undoped TiO_2_, T3: 3% Co-TiO_2_, T4: 5% Co-TiO_2_, T5: 7% Co-TiO_2_)

Sample	Lattice parameters						Cell volume	Positions					Agreement factors		
	*a* (Å)	*b* (Å)	*c* (Å)	*α*	*β*	*γ*		*x*	*y*	*z*	B_iso	Occ	*R*_p_	*R*_wp_	*χ* ^2^
T1	3.7925	3.7925	9.4860	90	90	90	136.44	0.00	0.75	0.125	4.18	1.00	2.90	3.80	1.55
Ti															
O								0.00	0.25	0.0891	−9.47	1.00			
T3	3.7924	3.7924	9.4925	90	90	90	136.53	0.00	0.75	0.125	6.00	0.97	2.08	2.67	1.07
Ti															
O								0.00	0.25	0.0872	−3.47	1.0			
Co								0.00	0.75	0.125	6.00	0.03			
T4	3.7958	3.7958	9.4960	90	90	90	136.82	0.00	0.75	0.125	0.21	0.95	2.19	2.75	1.09
Ti															
O								0.00	0.25	0.1070	0.76	1.00			
Co								0.00	0.75	0.125	0.21	0.05			
T5	3.7991	3.7991	9.4830	90	90	90	136.87	0.00	0.75	0.125	5.85	0.9	1.92	2.41	1.08
Ti															
O								0.00	0.25	0.0879	−5.44	1.00			
Co								0.00	0.75	0.125	5.85	0.1			

Raman spectroscopy has been used as an effective tool to study the crystallinity and disorder induced due to dopant incorporation in the host lattice, the presence of defects, *etc.* According to factor group analysis, anatase TiO_2_ having tetragonal structure has six Raman active modes (A_1g_ + 2B_1g_ + 3E_g_). Ohsaka^53^ studied Raman spectrum of an anatase single crystal, who investigated that the six allowed modes of anatase crystal appear at 144 cm^−1^ (E_g_), 197 cm^−1^ (E_g_), 399 cm^−1^ (B_1g_), 513 cm^−1^ (A_1g_), 519 cm^−1^ (B_1g_) and 639 cm^−1^ (E_g_). Fang *et al.*^54^ showed that E_g_ peak appears due to O–Ti–O symmetric stretching vibration in TiO_2_, B_1g_ appears due to O–Ti–O symmetric bending vibration and A_1g_ appears due to O–Ti–O anti-symmetric bending vibration.

**Fig. 4:**
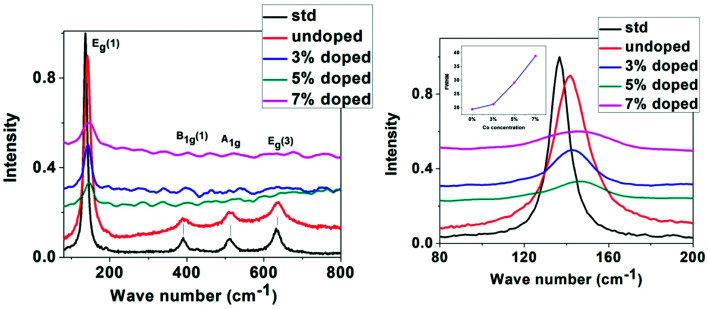
Left: Raman spectra of standard, undoped and Co-doped TiO_2_, right: expanded region of Raman spectra taken around 80–200 cm^−1^ (Inset showing the variation of FWHM with Co concentration).

Non-stoichiometry created due to oxygen vacancy and phonon confinement effect strongly affects the Raman spectrum producing shifting and broadening of some spectral peaks.^55–58^Fig. 4 shows the micro-Raman spectra of undoped and Co-doped TiO_2_ samples taken at room temperature in the range 80–800 cm^−1^. The spectrum of a standard powder sample taken from Sigma Aldrich is also given for comparison. The Raman lines at 142, 390, 511, 637 cm^−1^ can be assigned as E_g_, B_1g_, A_1g_, or B_1g_ and E_g_ modes of anatase phase respectively, the presence of which confirms that our samples belong to tetragonal anatase phase of TiO_2_. Four Raman modes have appeared and no mode corresponding to Rutile phase has been observed. In our study the most intense E_g_ (1) Raman mode at 142 cm^−1^ shows blue shift with doping and maximum blue shift is in 5% Co-doped sample. All the observed peaks show broadening and weakening of intensity as compared to undoped sample. The shifting of the position and broadening of the Raman peak has been explained due to phonon confinement effect due to nanoscale size of the crystallites.^58–60^

From XRD and TEM analysis crystallite size has been observed to be very small (6–15 nm). Also Raman signal of TiO_2_ is very sensitive to the vibrational mode of oxygen ions in the Ti–O bond.^61,62^ The presence of oxygen vacancy strongly influences the vibration of Ti–O which makes an effect on the intensity, position, and width of Raman signal. In our study it is observed that the E_g_ (1) peak has broadened as well as the intensity has decreased with increase in doping concentration while the intensity of other peaks has become almost negligible. The broadening of E_g_ (1) peak seen from the increase in FWHM with increase in Co concentration is indicated as an inset in the right Fig. 4. The shifting and broadening of the main E_g_ (1) Raman mode has been interpreted due to the nonstoichiometry in TiO_2_ lattice due to oxygen vacancies or disorder induced defects and phonon confinement effects. The crystallite size in the nanoscale range may affect the frequency shifting and broadening of Raman peaks due to the phonon confinement. The sudden reduction in scattering intensity in Co doped samples may be due to the breakdown of long-range translational crystal symmetry caused by the incorporated defects. The oxygen vacancies are introduced due to Co doping, which may be the reason for the observed weakening of the Raman signals.

**Fig. 5:**
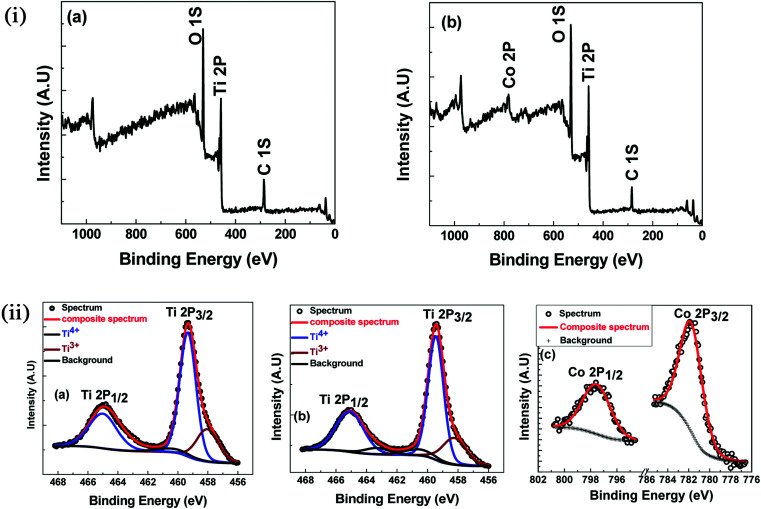
(i) XPS survey spectra (a) TiO_2_ (b) 7% Co-doped TiO_2_. (ii) XPS: high resolution scan (a) Ti 2p of TiO_2_ (b) Ti 2p of 7% Co-doped TiO_2_ (c) Co 2p of 7% Co-doped TiO_2_.

To further check the possibility of secondary phases, oxygen deficiency and oxidation states of Ti and Co in the near-surface region, XPS was carried out on undoped TiO_2_ and 7% Co-doped TiO_2_ samples at room temperature. The survey spectra of XPS are shown in the Fig. 5(i). Evidently, all the peaks can be ascribed to the elements Ti, C and O in undoped TiO_2_ while one extra peak of Co in addition to these peaks has appeared in 7% Co:TiO_2_ which is in good agreement with our expectation. The high-resolution spectra of Ti 2p and Co 2p were recorded and shown in the Fig. 5(ii). Fig. 5(ii)(a and b) shows deconvoluted XPS spectra of Ti 2p of undoped and 7% Co-doped TiO_2_ samples. The peak of Ti 2p_3/2_ for both the samples located at 459.49 eV corresponds to Ti^4+^. The broadening of Ti 2p_3/2_ obtained for both the samples at low binding energy could be ascribed to the appearance of Ti^3+^ or Ti^2+^, however, after deconvolution, the Ti 2p_3/2_ spectrum can be separated into two peaks which corresponds to Ti^4+^ and Ti^3+^. Furthermore, the Co peak appeared in 7% Co:TiO_2_ can be deconvoluted into two peaks one at 781.94 eV which corresponds to Co 2p_3/2_ and other at 797.6 eV corresponds to Co 2p_1/2_, in Co 2p spectra (Fig. 5(ii)(c)). The separation of Co 2p peak into 2p_3/2_ and 2p_1/2_ indicates that the valence state of Co is 2+. The spin–orbit splitting in our samples is approximately 16 eV. Broadening of the Raman modes and XPS data strongly supports the incorporation of Co^2+^ at the expense of Ti atoms in the host which creates oxygen vacancy. Confirmation of the presence of Ti^3+^ and Co^2+^ in the XPS analysis indicates that undoped and Co-doped samples possess certain amount of oxygen vacancies which corroborates the Raman spectra data.

**Fig. 6:**
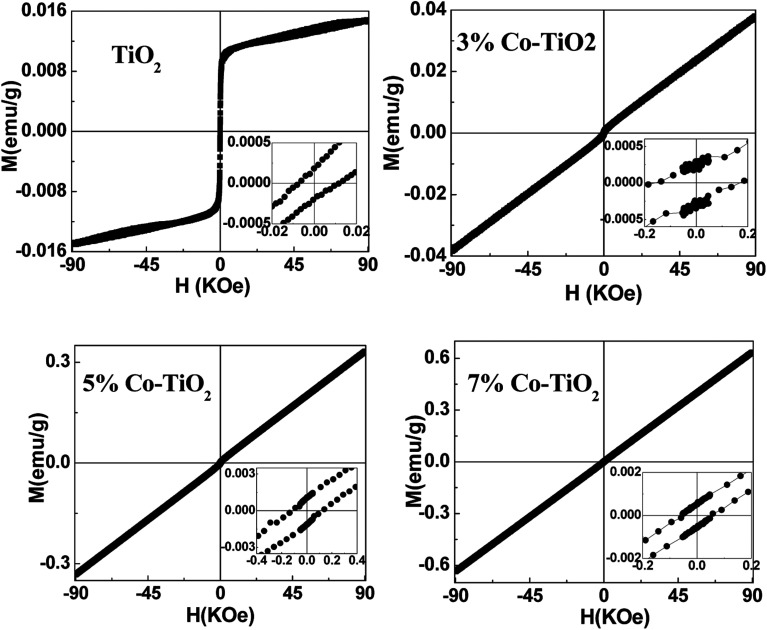
*M*–*H* plots taken at 300 K for all the samples of undoped and Co-doped TiO_2_ NPs. Insets in all the figures show expanded region at low field.

**Fig. 7:**
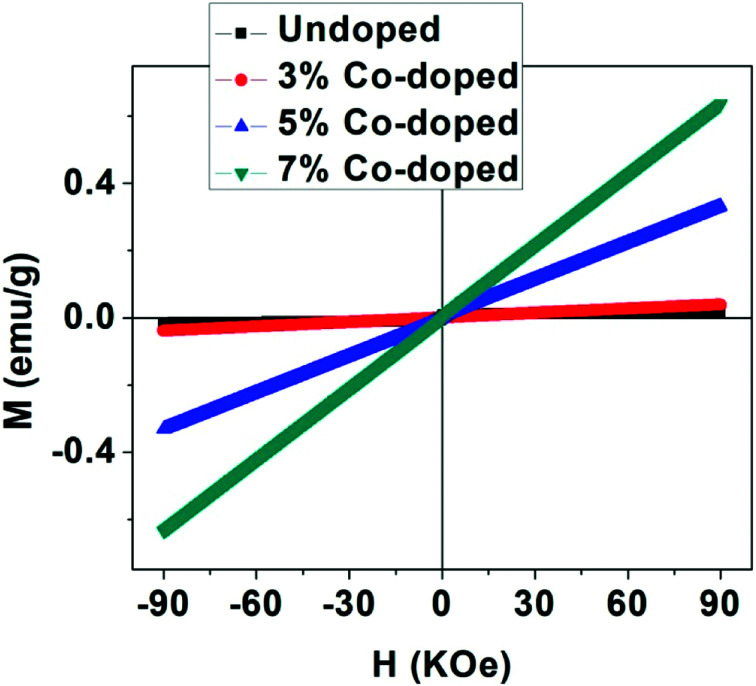
Magnetic behaviour (*M*–*H* plots) of undoped and Co-doped TiO_2_ NPs taken at 300 K.

To get information about the magnetic behavior of the undoped and Co-doped TiO_2_ powders, field dependent magnetization (*M*–*H*) measurements were carried out at 300 K with field varying from −90 kOe to +90 kOe. The *M*–*H* curves measured at 300 K show ferromagnetic behaviour (FM) of undoped TiO_2_ NPs which is shown in Fig. 6. However, the *M*–*H* curves of doped (3%, 5% and 10%) samples show paramagnetic (PM) type behaviour along with some FM ordering which is attested by the presence of hysteric nature at lower field region (shown as the inset of each figures). There is an increase in magnetic moment (emu g^−1^) with an increase in concentration of cobalt, and the saturation magnetization is not observed up to the maximum applied field of 90 kOe, as shown in Fig. 7. The magnetization values observed at 90 kOe are 0.013, 0.037, 0.330 and 0.636 emu g^−1^ for undoped, 3%, 5%, 7% Co-doped TiO_2_ nanoparticles respectively. Although square like hysteric behaviour has been obtained in undoped sample, the saturation could not be obtained even with the application of 90 kOe field which indicates the presence of paramagnetic (PM) kind of behaviour along with the FM. The presence of FM in undoped TiO_2_ suggests that Co doping is not only the origin of FM. The origin of weak FM in undoped TiO_2_ may be intrinsic *i.e.* due to presence of defects and/or oxygen vacancies which has been extensively reported. The oxygen vacancies at the surface of the nanoparticles may have introduced exchange interactions between localized electron spin moments which might have induced FM in undoped TiO_2_.^63^ The PM kind of behaviour may have originated duo to the formation of the clusters of oxides of Co, however, the same could not be detected in the XRD. It is to be noted that XRD plots of Co doped samples don't show any kind of impurity phases and this may be due to the lower detection limit of the instrument. It can be observed that the coercivity (*H*_c_) of doped samples is larger than that of undoped one (11 Oe), it is highest (170 Oe) in 3% doped sample, then decreased to 124 Oe for 5% and 54 Oe in 7% doped sample. The *M*_R_ value for undoped sample is 1.854 × 10^−4^ emu g^−1^ while for doped samples it is 2.8 × 10^−4^, 1.0 × 10^−3^ and 5.29 × 10^−4^ for 3%, 5% and 7% doped samples respectively. Surprisingly, both *H*_c_ and *M*_R_ values have been increased with Co doping but the trend is different. There is decrease in *H*_c_ with increase in Co concentration while the *M*_R_ value is highest in 5% doped samples. This can be explained on the basis of two types of interaction forces. The most prominent interaction among the clusters is the inter-particle dipole interaction. In addition, there also exists presence of inter-particle exchange interactions. Using Monte-Carlo (MC) simulations, Kechrakos and Trohidou *et al.*^64^ have investigated the role of inter-particle dipole and exchange interactions in determining the coercivity and remanence of the magnetization. The remanence increases due to the effect of weak exchange forces which favour ferromagnetic alignment of the moments. Dipolar interaction on the other hand produce a suppression of the remanence with concentration. The concentration dependence of remanence is determined by the competition between two types of interactions and a crossover occurs when the strength is comparable and in our case it may have happened at 5% concentration after which the remanence is decreased. It is also explained that^64^ when both types of interactions are present, the coercivity decreases with concentration for all values of exchange strength.

From this it can be inferred that magnetism in Co-doped samples is mainly due to Co doping. But in this study oxygen vacancies play an important role in enhancing the magnetism. From XRD and XPS no secondary phases of either Co metal or Co oxide related phases have been detected and both XPS and Raman spectra indicate presence of more oxygen vacancies in doped samples. The enhancement in the magnetic moment (as can be seen from Fig. 7) may be due to exchange interaction between sp-bond electrons or holes and the d-electron spins localized at the magnetic ions,^65^ superexchange between complexes (oxygen vacancies + magnetic impurities) which are stabilized by the electron transfer from vacancies to impurities^66^ and magnetic polaron formed by trapped electrons in oxygen vacancies and magnetic ions around it.^67^ Thus we can make a conclusion that oxygen vacancies play an important role for FM ordering and the doping enhances the FM ordering. The observed magnetism is purely intrinsic property in undoped and both extrinsic and intrinsic properties account for the magnetism in Co-doped TiO_2_ nanoparticles.

## 4. Conclusion

Influence of cobalt doping on the structural and magnetic properties of TiO_2_ nanoparticles prepared by a simple wet chemical method was investigated. The structural analyses showed that the synthesized samples were in anatase phase with slight deviation in lattice variation which were due to Co doping. The morphological study by scanning electron microscope and transmission electron microscope indicate the formation of nanoparticles of sizes 6–10 nm. High resolution TEM image shows clear lattice fringes indicating highly crystalline nature of the nanoparticles which was further analysed by selected area electron diffraction pattern which indicates polycrystalline nature of anatase TiO_2_. The shifting and broadening of most intense E_g_ (1) mode in micro-Raman study of Co-doped TiO_2_ nanoparticles indicate the incorporation of Co in TiO_2_. Presence of oxygen vacancies in undoped and Co-doped TiO_2_ samples is evident from the X-ray photoelectron spectra and Raman spectra analysis. The magnetic measurement shows ferromagnetic behavior at room temperature in undoped TiO_2_ which has originated due to the presence of oxygen vacancies which is intrinsic in nature. But *M*–*H* curve of Co-doped TiO_2_ shows coexistence of ferromagnetic and paramagnetic phases. The ferromagnetism has arisen due to oxygen vacancies and the enhancement in magnetism is due to Co doping and the paramagnetism may be due to presence of some undetected clusters of oxides of cobalt.

## Conflicts of interest

There are no conflicts of interest to declare.

## Supplementary Material
